# Creatine kinase B deficient neurons exhibit an increased fraction of motile mitochondria

**DOI:** 10.1186/1471-2202-9-73

**Published:** 2008-07-28

**Authors:** Jan WP Kuiper, Frank TJJ Oerlemans, Jack AM Fransen, Bé Wieringa

**Affiliations:** 1 Department of Cell Biology, Radboud University Nijmegen Medical Centre, P.O. Box 9101, 6500 HB Nijmegen, The Netherlands

## Abstract

**Background:**

Neurons require an elaborate system of intracellular transport to distribute cargo throughout axonal and dendritic projections. Active anterograde and retrograde transport of mitochondria serves in local energy distribution, but at the same time also requires input of ATP. Here we studied whether brain-type creatine kinase (CK-B), a key enzyme for high-energy phosphoryl transfer between ATP and CrP in brain, has an intermediary role in the reciprocal coordination between mitochondrial motility and energy distribution. Therefore, we analysed the impact of brain-type creatine kinase (CK-B) deficiency on transport activity and velocity of mitochondria in primary murine neurons and made a comparison to the fate of amyloid precursor protein (APP) cargo in these cells, using live cell imaging.

**Results:**

Comparison of average and maximum transport velocities and global transport activity showed that CK-B deficiency had no effect on speed of movement of mitochondria or APP cargo, but that the fraction of motile mitochondria was significantly increased by 36% in neurons derived from CK-B knockout mice. The percentage of motile APP vesicles was not altered.

**Conclusion:**

CK-B activity does not directly couple to motor protein activity but cells without the enzyme increase the number of motile mitochondria, possibly as an adaptational strategy aimed to enhance mitochondrial distribution versatility in order to compensate for loss of efficiency in the cellular network for ATP distribution.

## Background

Neurons, by virtue of their unique architecture, have developed specific transport systems to regulate anterograde and retrograde flow of macromolecules, vesicles or organelles between the cell body and distal regions in the axon and dendrites. To maintain efficiency and directionality in the bidirectional flow of these cellular constituents strict control over movement of cargo by motor proteins on cytoskeletal elements such as microtubules, intermediate filaments, and actin, is needed [1-3]. One of the basic elements in this control is adequate fuelling with ATP, the major carrier of cellular energy. Homeostasis of global and compartmentalized ATP levels, i.e. regulation of production, distribution, and consumption of intracellular ATP, is controlled by an elaborate metabolic network, which varies with cell type. In neurons this circuit involves both cytosolic-glycolytic and oxidative mitochondrial production pathways and a high level of ATP consumption for fuelling of acto-myosin dynamics, ion transporters, and neurotransmitter cycling activity in the synapse [4-6]. Neurons use glucose from the circulation as the main carbohydrate source for ATP production, but – depending on specific physiological conditions – a fair percentage of their energy may also be derived from lactate, which they exchange with astrocytes [7,8], or from ketone bodies imported from circulation. Because of the highly branched morphology of neurons, sites of energy consumption are usually spatially separated from sites of energy generation in this cell type. Since diffusion of ATP might usually be too slow to achieve optimal supply of high-energy phosphoryl groups (~P), neurons have developed more efficient mechanisms for transport and distribution of ~P. One way to minimize the diffusion distance of ATP and regulating natural inhomogeneity in ATPs intracellular distribution is by redirecting mitochondria to sites were ATP demand is high, e.g. in the vicinity of synapses [9]. This requires active mitochondrial transport, which is mainly driven by members of the kinesin and dynein superfamilies of microtubule directed motor proteins such as KIF1Bα and KIF5 [10,11], although, actin guided motility may also be involved [12-14].

An alternative strategy to optimize spatial energy transfer within cells is to relay high-energy phosphoryl groups (~P) by enzymatic transfer systems, such as the creatine kinase (CK) family of isozymes [15]. These enzymes buffer ATP and ADP levels by the reversible transfer of ~P onto creatine (Cr) (MgATP^2- ^+ Cr ↔ MgADP^- ^+ CrP^2- ^+ H^+^) [15,16]. Indeed, CKs are mainly expressed in tissues with high energy-turnover and sudden rises in energy demand, such as muscle and brain [17,18]. Ubiquitous mitochondrial CK (UbCKmit) and cytosolic brain-type CK (CK-B) are the two predominant isoforms in brain [17,19] and broadly distributed throughout neurons (moderate-low expression) and glial cells (high expression in astrocytes and microglia) across different brain areas. The CK system provides cells with both a temporal and spatial energy buffer [15,16]. During transient rises in energy consumption, the CrP pool is addressed by CK to provide the cell with ATP [18,20]. In addition, CK isozymes connect spatially separated subcellular locales of ATP generation and ATP hydrolysis [21,22]

We have demonstrated that genetic ablation of CK-B in mice causes changes in behavior, diminished performance in spatial learning tasks and delayed development of pentylenetetrazole-induced seizures [17]. Furthermore, the intra- and infrapyramidal mossy fiber areas in CK-B-/- mice appeared increased. We explained these features by diminished synaptic plasticity or compensatory adaptation with altered neuronal outgrowth during development.

Here we investigated whether compromised intracellular energy transport could underlie the diminished synaptic plasticity or altered morphology (in analogy to [23-25]).

Intracellular transport in neurons is comprised of membranous organelles and cytoplasmic proteins (or protein complexes) that are conveyed from the cell body to the synapse, and vice versa, by either fast or slow axonal transport [1]. In general, movement of organelles is mediated by fast axonal transport, whereas cytosolic and cytoskeletal proteins move at a slower pace. This difference in velocity is attributed to the duty ratio of the motor proteins involved in both types of transport [1,26].

CK-B was identified in slow component B (SCb) which, together with slow component A (SCa), make up the branch of slow axonal transport [27]. However, it is not known if CK-B facilitates this particular type of axonal transport, or that it is merely transported as inert cargo to subcellular destinations where it is needed. To address the question whether CK-B enzymatically contributes to axonal transport in more detail, we compared cultured primary hippocampal neurons derived from CK-B knockout and wildtype mice and monitored the fate of YFP tagged amyloid precursor protein (APP) as a representative component in fast transport. This type of transport correlates with a high duty ratio of motor proteins and with high ATPase activity. In addition, we analysed mitochondrial dynamics. Our results suggest that CK-B does not influence the velocity of intracellular transport of APP or mitochondria in neurons. Rather, cells deficient in CK-B display show a conspicuous alteration in magnitude of transport, concomitant with an increase in the fraction of motile mitochondria.

## Results

### Distribution of CK-B in primary neurons

To assess effects of CK-B efficiency in primary neurons, we used a co-culture system of hippocampus-derived neurons on a monolayer of primary astrocytes (see material and methods). Neuronal expression of CK-B has been demonstrated in several organisms, but not much is known about its subcellular localization [28,29].

**Figure 1:**
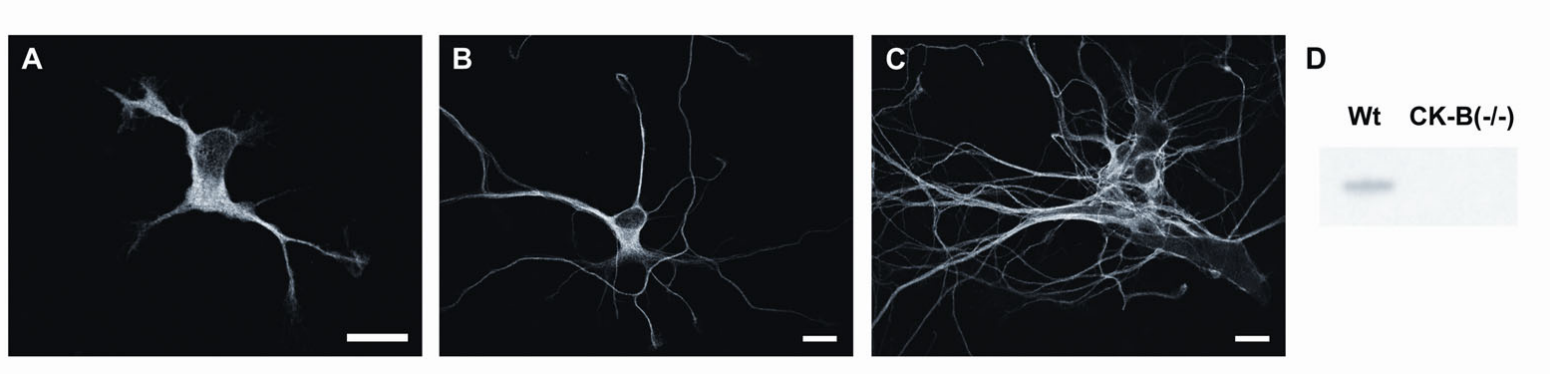
Primary neurons derived from wildtype mice were co-cultured with astrocytes for 1, 3 and 6 days. Subsequently, the cells were fixed and immunostained with an isoform specific monoclonal antibody against CK-B (21E10). Confocal images display the subcellular distribution of CK-B after **A) **1 day, **B) **3 days and **C) **6 days. Image bars represent 10 μm. **D) **Lysates were prepared from wildtype and CK-B deficient neurons (7 div) and zymogram analysis was applied to determine enzymatic CK activity.

Immunolocalization on murine hippocampal neurons of different age with an isoform-specific antibody [30] showed that CK-B was evenly distributed throughout the entire cell body, and rarely detected in the nucleus. Figure 1 displays confocal images of hippocampal neurons, which were cultured for 1, 3 or 6 days *in vitro *(Figure 1a,b and 1c, respectively). No obvious changes were observed in either the intensity or localization of CK-B during the 6-day culture period.

Neurons derived from CK-B knockout mice did not display any positive immunostaining with our antibody, demonstrating specificity of our assay (data not shown). In addition to localization studies, we also performed zymogram analysis on cultured primary neurons to assess enzymatic activity. As expected, CK-B catalytic activity was present in wildtype cells, but was completely absent in CK-B knockout cells (Figure 1d).

### APP-transport in CK-B deficient neurons

**Figure 2:**
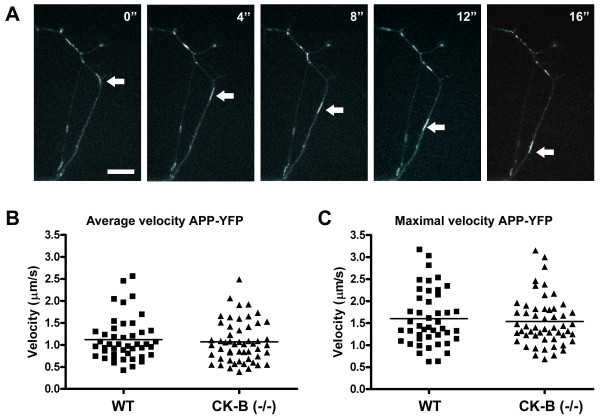
7-day-old CK-B deficient and wildtype neurons were transfected with plasmid DNA encoding APP-YFP. After 24 hours cells were subjected to live imaging at 37°C and 5% CO_2_. Panel **A **shows 5 successive captures of a transfected neuron. An APP-YFP-containing vesicle, which traverses the neuron, is marked by arrows. The bar represents 10 μm. **B) **The average velocity of APP-YFP positive vesicles in both wildtype and CK-B deficient neurons was calculated and plotted in the diagram. For wildtype and CK-B (-/-) cells 45 vesicles and 53 vesicles were tracked, respectively. **C) **Maximum velocities for individual particles of wildtype and CK-B knockout cells were also plotted.

To investigate a possible role for CK-B in axonal transport, we focused on the amyloid precursor protein (APP). APP is a membrane spanning type-1 protein which is conveyed from the cell body to the synapse by fast axonal transport [31-33]. The kinesin KIF-I was identified as the tubulin directed motor protein responsible for APP transport [33] and real time live imaging revealed that APP fused to Yellow Fluorescent Protein (YFP) is transported over long distances with speeds up to 9 μm/s [32]. To maintain this dynamics a continuously high supply of ATP is needed for motor protein functioning. To test whether CK-B mediated ~P transfer has a role in safeguarding this process, we compared the appearance and movements of APP containing vesicles in wildtype and CK-B knockout neurons after transfection with YFP-tagged APP. In Figure 2a we show 5 successive frames of a time-lapse registration of a cell with APP-YFP. Careful analysis demonstrated that APP was transported in elongated tubular vesicles, confirming earlier observations by Kaether *et al. *[32]. Vesicle appearance did not overtly differ between wildtype and CK-B knockout cells. For further comparison, 45 and 53 APP-YFP containing vesicles from wildtype and knockout cells, respectively, were tracked and their average velocities calculated. Figure 2b shows that the distribution of velocities was similar for both types of cells. On average, APP-YFP vesicles moved at 1.12 ± 0.49 and 1.07 ± 0.47 μm/s for wildtype and CK-B knockout, respectively. Because vesicles sometimes changed their speed during time-lapse recording, we also calculated the maximum velocity for each vesicle during one recording. In figure 2c these maximum values are displayed. Maximal velocities found were comparable, with 1.60 ± 0.62 μm/s for APP-YFP vesicles in wildtype cells and 1.54 ± 0.55 μm/s for CK-B knockout cells. Also no difference was found in the distribution of maximal velocities. Both knockout and wildtype vesicles reach maximal velocities up to 3 μm/s. It may be of note here, that this is 3 times slower than the maximum and 4 times slower than the average velocities of APP reported in rat primary neurons [32].

### Mitochondrial transport in CK-B deficient neurons

**Figure 3:**
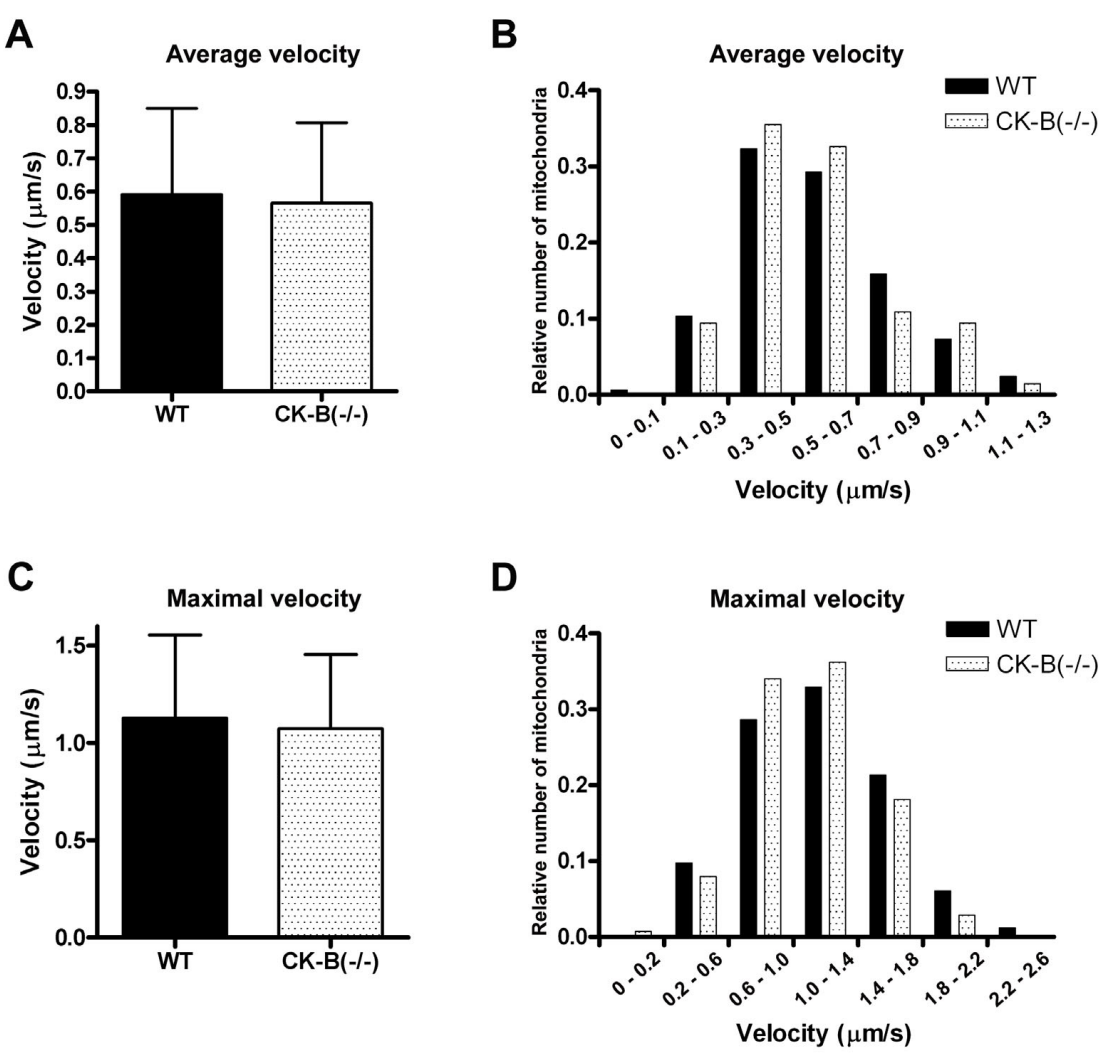
Mitochondria of 7-day-old CK-B deficient and wildtype neurons were stained with rhodamine 123. Live imaging was applied to track these mitochondria. Average velocities were determined for mitochondria of wildtype cells (164 mitochondria) and CK-B deficient cells (138 mitochondria). The bar diagrams in panel **A**, display average velocities with error bars representing the standard deviation (SD). Panel **B **shows the distribution of mitochondrial velocities. Maximum velocities for individual tracked mitochondria were also determined and the average of these are presented in panel **C**. Error bars represent SD. **D**) The distribution of maximal velocities of individual mitochondria are shown for wildtype and CK-B knockout neurons.

Mitochondrial transport and repositioning is an important mechanism for neurons to comply with alterations in local energy demand. Fission, fusion and intracellular motility are essential processes involved in the regulation of subcellular distribution of mitochondria. It is therefore not surprising that many neurodegenerative diseases are associated with perturbations of these processes [2,34]. To investigate if CK-B coordinates the fueling role and transport fate of mitochondria, we compared the dynamic behavior of mitochondria between primary hippocampal neurons from wildtype and CK-B knockout mice. Staining with rhodamine 123 to visualize mitochondria and time-lapse recording and subsequent image analysis were used to determine the average mitochondrial velocity (Figure 3a). Mitochondria included in the analysis traveled a minimum of 3 frames and were tracked till they stopped or reversed direction. Mitochondria from wildtype cells traveled at an average velocity of 0.59 ± 0.26 μm/s, which was almost identical to CK-B knockout cells (0.57 ± 0.24 μm/s). Furthermore, frequency histogram analysis revealed no differences in the distribution of average mitochondrial velocity (Figure 3b), suggesting that there is also no subset of mitochondrial movements that is affected by CK-B deficiency. Because a single mitochondrion could vary its speed during the cause of one movement, we also calculated the maximum speed for every mitochondrion during its recording period. The bar-diagram in figure 3c displays the average of maximal reached speeds of all tracked mitochondria in wildtype (1.13 ± 0.43 μm/s) and CK-B deficient cells (1.07 ± 0.38 μm/s). A frequency distribution diagram of maximal speeds also revealed no significant differences in maximal velocities of mitochondria between knockout and wildtype cells (figure 3d).

**Figure 4:**
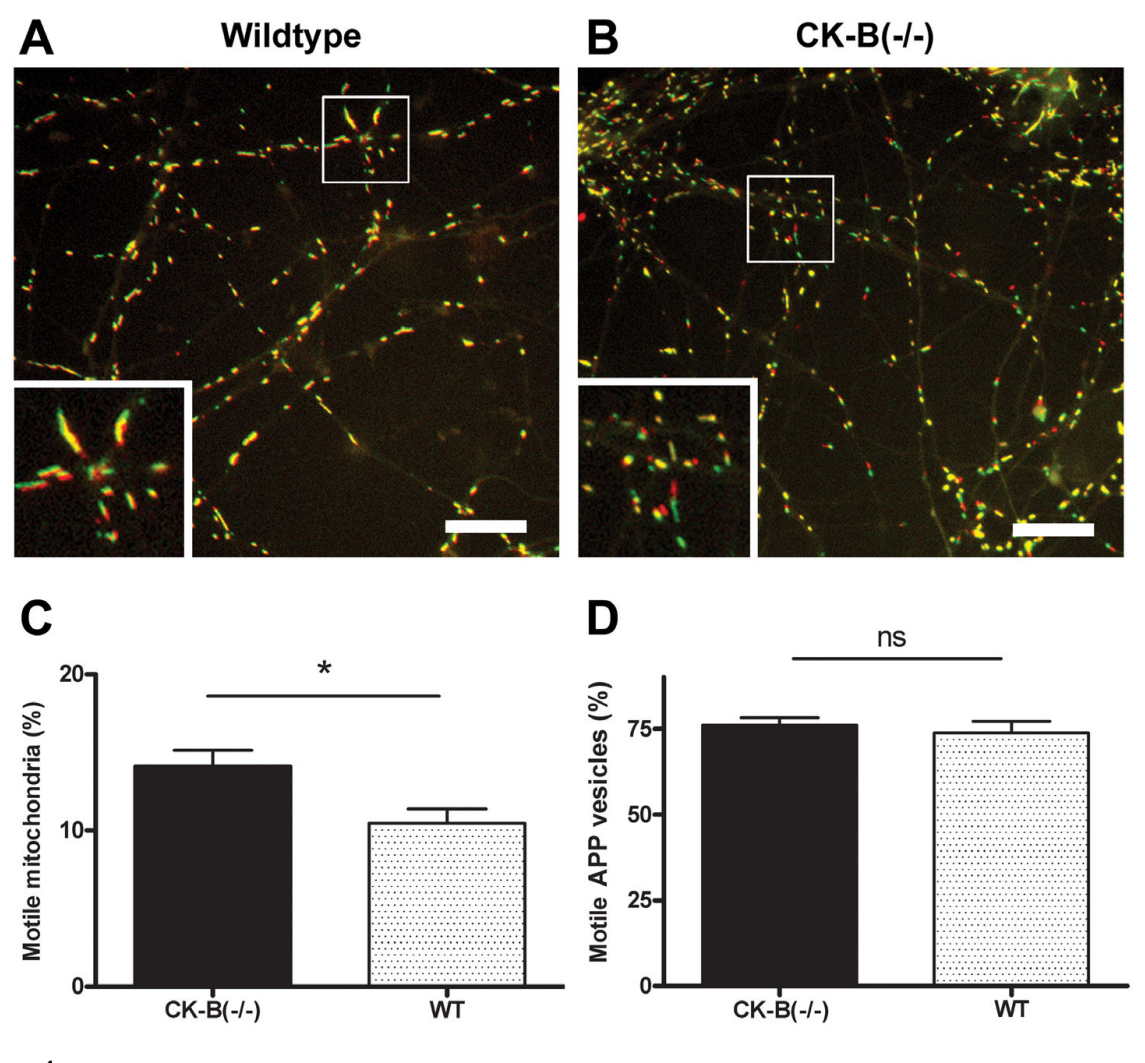
The percentage of motile mitochondria in wildtype and CK-B knockout neurons was determined. The first (red) frame and the 100^th ^frame (green) of a movie were merged. Yellow mitochondria are non-motile mitochondria. Panel **A) **1^st ^and 100^th ^frames of a movie of mitochondria in a wildtype neuron after merging. Panel **B) **displays the CK-B knockout equivalent. For wildtype (n = 12) and CK-B knockout (n = 15) movies were analyzed using the stack difference method (see methods). The percentage of motile mitochondria is presented in the scatter dot plot in panel **C **(p < 0.05). The percentage of motile YFP-APP particles in wildtype and CK-B knockout neurons was determined by analyzing 2 sets of merged frames from every movie (see also Additional Files 1 and 2). Panel **D **shows the average percentages (WT: 74%; CK-B: 76%; p = 0.57) of motile YFP-APP vesicles (error bars represent SEM). n = 22 for WT and n = 14 for CK-B(-/-).

Since CK-B deficiency had no impact on the average and maximal velocities by which mitochondria travel in neurites, we wondered if lack of CK-B mediated ~P transfer could elicit more subtle effects and have impact on the rate of engagement in intracellular transport or affect the process of anchorage of mitochondria, two types of events which are also believed to be regulated by local energy demand [35,36]. To answer this question, we analysed whether the fraction of mitochondria that was rendered motile might be affected by CK-B deficiency (Figure 4). Image stack difference analysis was applied to estimate the percentage of mitochondria that moved during the time of one recording (3 minutes). For wildtype cells 10.5 ± 3.2% of mitochondria were motile at any moment during the course of one recording. Surprisingly, CK-B deficient cells showed a significant increase of 35% (p < 0.05) in motile mitochondria (14.1 ± 3.8%). The dot plot in figure 4c also clearly shows the shift towards more motile mitochondria in CK-B deficient cells. To validate this conclusion, we also applied a method with manual counting (see Additional file 1). Importantly, the outcome of this analysis was almost identical (37% increase in the fraction of motile mitochondria in CK-B deficient neurons), although absolute percentages of motile mitochondria were lower with this method (5.9% ± 2.2% for wildtype and 8.1 ± 2.0% for CK-B(-/-); p < 0.0005) (see figure in Additional file 2). When this same image analysis methodology was applied to YFP-APP vesicles, no significant effect of absence or presence of CK-B was found (Figure 4d).

In conclusion, CK-B is not influencing the speed of intracellular transport of APP or mitochondria once this transport is initiated, however, in cells that lack CK-B a larger fraction of mitochondria is recruited into the actual motile pool.

## Discussion

ATP generation and distribution is essential for the highly compartmentalized eukaryotic cell. Especially in neurons with their extended axonal and dendritic networks, it is important to modulate fuelling logistics. Spatially confined cellular processes like synapse functioning require local supply of energy. In order to fuel these functions optimally, they are coupled to sites of energy production. The CK system provides cells with a temporal and spatial ATP buffer to connect local energy consumption with sites of energy production [16,28,37]. Other phosphotransfer enzymes, like adenylate kinases and nucleoside diphosphate kinases are also active in neurons, and serve in the global network that distributes ATP throughout the cell. In addition, neurons have the capacity to relocate their energy production machinery to specialized subcellular sites to help in local ATP generation. Partitioning of glycolytic enzymes on cortical actin or membrane-near sites can provide local energy to membrane pumps by functional coupling [38,39], whereas oxidative generated ATP can be generated locally, by recruiting mitochondria to sites of high ATP consumption such as synapses and dendritic spines [9,35,40].

We focused on the question whether CK-B facilitates efficient axonal transport by comparing transport of APP and mitochondria in primary murine neurons derived from CK-B deficient and proficient mice.

Active transport of cargo in neurons is achieved by a wide variety of motor proteins, which are guided by the infrastructure of the cellular cytoskeleton [14]. These cargos can travel along actin filaments or microtubules either by plus end or minus end directed trafficing, thus facilitating both anterograde and retrograde axonal transport. Both types of cytoskeletal structures have their own assortment of motor proteins, which can be divided in actin-guided myosins [41] and microtubule-guided kinesins and dyneins [3]. A common denominator for myosins, dyneins and kinesins is that they require ATP hydrolysis to exert their function.

Fast axonal transport of membranous organelles and membrane proteins depends on highly processive motor activity and, consequently, a steady and adequate ATP/ADP ratio for optimal fuelling of motor proteins. Our data show that the actual speed of fast axonal transport of APP-YFP is not affected by CK-B deficiency. Although the maximum and average velocities observed are lower than reported for rat neurons [32], this may be a mouse related feature and no differences between wildtype cells and CK-B deficient cells were found. In addition, mitochondrial transport velocities were not affected in CK-B knockout cells. Mitochondria are subject to saltatory movement, which involves cycles of anterograde and retrograde transport driven by kinesins and dyneins, respectively [40]. Because axons and dendrites in 7-day-old cultures of primary neurons are totally intertwined, we were unable to distinguish between axonal/dendritic or anterograde/retrograde transport. Therefore, and also because other groups have reported that mitochondrial velocity and the rate of anterograde and retrograde transport are highly similar in axons and dendrites of hippocampal neurons [42,43], we decided not to discriminate between CK-B effects further. At this point, we thus consider it unlikely – but can also not fully exclude – that CK-B deficiency affects the ratio of anterograde/retrograde transport of mitochondria.

We hypothesized that CK-B deficiency would bring about an altered capacity to distribute intracellular ATP, and create abnormal inhomogeneity in local ATP. Because neurons rearrange their mitochondria according to local ATP needs [9,40], altered local ATP distribution may determine altered mitochondrial motility. The fact that no differences in either mitochondrial or YFP-APP velocities in combined anterograde/retrograde transport were found is therefore an interesting finding in its own right. Possibly, flexibility in the energetic network, with higher ~P flux through adenylate kinase (AK) or glycolytic enzymes helps to compensate the loss of CK-B [44-46], or – alternatively – mitochondria produce are still able to produce enough ATP to sustain their own transport.

Indeed, our findings suggest that initiation or abrogation of transport may be steps in the process that are more crucially dependent on cell energy state. Quantification of the fraction of mobile mitochondria revealed that CK-B deficient neurons contain on average 36% more mitochondria in the motile fraction. The metabolic factors that modulate and mobilize mitochondrial motility are largely unknown. Local ATP depletion, or locally elevated H^+ ^and ADP levels caused by CK absence, could serve as a direct or indirect signal to attract mitochondria, or arrest nearby motor activity, arresting mitochondria while passing the "fuel-arid" area [40,47-49]. A combination of mechanistic events is also possible. In addition, secondary effects like inadequate Ca^2+ ^handling, due to CK-B deficiency [20,50], could act in signalling pathways for mitochondrial motility and/or docking [51]. For neurons, it has been found that local neuronal growth factor (NGF) application triggers mitochondrial recruitment through PI3K. Moreover, an intact F-actin cytoskeleton is required [52,53], which is organized by the action of formins and RhoA [54]. Interestingly, we recently found that CK-B increases the F-actin content in phagosomes [55]. Although the underlying molecular mechanisms of this effect on F-actin are yet unclear, it is tempting to speculate that CK-B deficiency in neurons could induce less efficient actin accumulation at sites of mitochondrial arrest. Indeed, a prominent role for actin-state in mitochondrial movement has been proposed [56]. Future research might help to discriminate between these different putative mechanisms.

## Conclusion

We conclude that different types of axonal and dendritic transport in neurons do not directly require ATP generated by CK-B. However, CK-B mediated phosphotransfer is functionally interconnected to events that determine the transport-initiation or -docking efficiency of mitochondria in neurons.

## Methods

### Isolation and culture of primary neurons

The generation of CK-B knockout mice and the study of genotype-phenotype relationships of these animals in comparison to wildtype controls has been described in detail elsewhere (also [17,57]). Primary cultures of mouse hippocampal neurons were established using a modified protocol [58]. In short, brains were isolated from CK-B(-/-) [17] fetuses (E16.5) or fetuses of mixed background (C57BL/6 × 129Ola). Meninges were removed and hippocampi were separated from the hemispheres. Hippocampi were incubated for 20 minutes at 37°C in Hanks' Balanced Saline Solution (HBSS, Gibco) containing 0.05% trypsin, 1 mM EDTA and 20 mM HEPES (pH 7.35) and subsequently dissociated by pipetting and seeded onto 24 mm coverslips. Cells were allowed to attach for 3–4 hours in Neurobasal medium (Gibco), after which they were placed inverted on a layer of primary astrocytes (also see [58]). The co-culture was maintained in Neurobasal medium containing 1× B27 supplement (Gibco), 0.5 mM glutamine and 0.05 mg/ml gentamycin (= NBM+).

### Creatine kinase activity (zymogram)

Cultured primary neurons (5 days *in vitro*) were lysed in buffer containing 12.6 mM Na_2_HPO_4_, 2.8 mM KH_2_PO_4_, 0.05% Triton-x-100 and 0.3 mM DTT. Zymogram analysis was performed as described [20] and, zymograms were subsequently developed using the colorimetric detection kit from Sigma Diagnostics (procedure number 715-EP).

### Indirect immunofluorescence

Neurons (3–7 days *in vitro*) grown on glass coverslips were fixed with 2% paraformaldehyde in PHEM buffer (25 mM HEPES, 10 mM EGTA, 60 mM PIPES, 2 mM MgCl_2_, pH 6.9), permeabilized with 0.1% Triton X-100 and incubated 20 min in PBS containing 4% bovine serum albumine (BSA). CK-B was detected by subsequent incubation of monoclonal 21E10 (1:2000) [30] and goat-anti-mouse IgG conjugated to Alexa Fluor 488 (Molecular Probes). Images were taken with a Biorad MRC1024 confocal microscope using an oil immersion 60× objective.

### Transfection and rhodamine 123 labeling of neurons

Neurons (7 days *in vitro*) grown in glass bottomed 35 mm Willco dishes (GWSt-3522) were transfected using Nupherin-neuron (Biomol) transfection reagent in combination with Lipofectamine (Invitrogen). Per dish 0.5 μg pcDNA3-APP-YFP (kind gift from Carlos Dotti [32]) and 2.5 μl Nupherin were premixed in phenol red free Neurobasal medium and incubated for 10 minutes. An equal volume of phenol red free NBM with 1 μl Lipofectamine was added and after 30 minutes this mix was added to the neurons. After 2 hours the medium was replaced by NBM+ medium and neurons were cultured for 24 hours prior imaging. For tracking mitochondria cells were loaded with rhodamine 123 (10 μM) for 1 minute in NBM+ w/o phenol red.

### Live imaging and image analysis

Cells cultured on Willco dishes were imaged on an inverted microscope (Axiovert 200 M; Zeiss, Jena, Germany) equipped with a temperature controlled CO_2 _incubator (type S) and sample stage, and using a PlanApochromatic 63 × 1.4 oil immersion Plan NeoFluar DIC lens (Carl Zeiss GmbH, Jena, Germany). Rhodamine 123 was excited using a monochromator (Polychrome IV; TILL Photonics, Gräfelfing, Germany) set at 488 nm. Images were recorded with the appropriate filter set (Omega Optical, Brattleboro, VT, USA) on a CoolSNAP HQ monochrome charge-coupled device (CCD) camera (Roper Scientific, Vianen, The Netherlands). All hardware was controlled with Metafluor 6 software (Molecular Devices Corp., Downingtown, PA, USA).

For particle (i.e. APP-cargo vesicles or mitochondria) tracing, sequential images were taken every 2 seconds to obtain image stacks of 100 images each. Particles were tracked using Metamorph 6 software (Molecular Devices Corp., Downingtown, PA, USA) software by marking them manually in subsequent frames (only particles that moved at least in 3 subsequent frames were tracked). The velocity per particle-vesicle was calculated by dividing the travelled distance by time. Additionally, for each moving particle the maximal velocity (during two subsequent frames) was determined. The number of analysed particles is mentioned in the text or legends.

To estimate the percentage moving mitochondria the same dataset as mentioned above was analyzed with ImageJ software version 1.34s (U. S. National Institutes of Health, Bethesda, Maryland, USA, ). The original image stacks were converted to binary stacks by manually applying a threshold, according to the quality of each individual stack. The total number of mitochondria in the stack was counted using the particle count function (5 < mitochondria <50 pixels). To distinguish between moving and stationary mitochondria, every frame "n" was compared to an earlier frame "n-3" ("stackdifference" option from the ImageJ kymograph plugin, EMBL, Heidelberg, Germany). The resulting stack of images contains motile mitochondria, which were counted. Numbers obtained were divided by two to compensate for double counting of both "old and new" mitochondrial positions in "difference-stack" images. Finally, percentages of motile mitochondria in the stacks were calculated. For more information see Additional file 1.

## Authors' contributions

BW was responsible for project planning. JWPK, JAMF and BW conceived and designed the experiments. JWPK performed most of the experiments. JAMF contributed to microscopic analyses. FTJJO contributed methodology and performed experiments. JWPK and BW wrote the paper. All authors read and corrected the paper and added suggestions.

## Competing interests

The authors of this manuscript declare that they have no competing interests.
